# Evaluation of a pilot family planning educational seminar and subsequent attitudes towards family planning among Muslim communities in Tanzania

**DOI:** 10.1371/journal.pone.0315410

**Published:** 2025-02-07

**Authors:** Alexandra A. Cordeiro, Hajirah Gumanneh, Aneth Nzali, Valencia J. Lambert, Amina Yussuph, Albert Kihunrwa, Yassin Mchondo, Ramadhan Mtita, Hidaya Yahaya, Samuel E. Kalluvya, Joyce Wamoyi, Mehrunisha Suleman, Jennifer A. Downs, Agrey H. Mwakisole

**Affiliations:** 1 Center for Global Health, Weill Cornell Medicine, New York, New York, United States of America; 2 Weill Cornell Medical College, New York, New York, United States of America; 3 Mwanza Intervention Trials Unit, MITU, Ilemela, Mwanza, Tanzania; 4 Bugando Medical Centre, Mwanza, Tanzania; 5 Tablighi na Dawaa Bakwata, Mwanza, Tanzania; 6 Nyamagana District Hospital, Mwanza, Tanzania; 7 National Institute for Medical Research, Mwanza, Tanzania; 8 Ethox Center, Nuffield Department of Population Health, University of Oxford, Oxford, United Kingdom; 9 Global Studies Center, Gulf University for Science and Technology, Mubarak Al-Abdullah, Kuwait; 10 Mwanza Christian College, Mwanza, Tanzania; PLOS: Public Library of Science, UNITED KINGDOM OF GREAT BRITAIN AND NORTHERN IRELAND

## Abstract

Evidence has demonstrated that uncertainty about compatibility with religious beliefs and limited health knowledge hinder uptake of family planning (FP), even among women who would like to prevent or delay childbearing. Empowering women and men to choose the number and timing of children is a global goal and enhances both maternal and child health. Building on data demonstrating the effectiveness of religious leaders in Tanzania to provide public health information in communities, the aim of this study was to understand whether and how an educational seminar about FP that was provided to Tanzanian Muslim religious leaders could be an effective means by which education about FP could reach members of their communities. This study employed a mixed-methods approach to pilot-test a one-day educational seminar about social, medical, and theological aspects of FP. The seminar was provided to Muslim religious leaders from two mosques in northwest Tanzania in April 2022. Six weeks after the seminar, the same religious leaders were invited to evaluate the seminar both by a quantitative survey assessing acceptability, appropriateness, and feasibility, and in in-depth interviews. Interviews explored participants’ knowledge and perceptions of FP, views of its permissibility in Islam, and actions that they had taken since attending the seminar. Demographic and survey data was analyzed using R software. Thematic analysis using de-identified transcripts was performed using NVivo (Version 12). In June 2022, 48 Muslim religious leaders (26 women; 22 men) completed the quantitative survey and in-depth interviews. Participants rated the seminar as highly acceptable, appropriate, and feasible, with mean scores above 4.5 out of 5 for every statement. Participants viewed the seminar as enlightening and expressed that it improved their knowledge about FP and enabled them to consider FP from both medical and Islamic faith-based viewpoints. Others described having taught their communities about FP and described the positive impact the seminar had for enhancing couple communication and enabling FP uptake for those desiring to use it. Almost all participants recommended that the seminars return to their community more frequently and indicated the importance of allowing discussion time for men and women separately during part of the seminar. Muslim religious leaders reported feeling equipped by an educational seminar to teach about FP to their communities. These data highlight the high potential of trusted religious leaders to build knowledge about FP, which could address women’s current unsatisfied demand for FP and promote maternal and child health in Muslim communities in Tanzania.

## Background

Pregnancies that occur too early or too often contribute to high rates of maternal and newborn morbidity and mortality, particularly in low- and middle-income countries [1]. Family planning (FP) can support adequately spaced pregnancies, promote women’s and children’s health, and encourage women’s empowerment by enabling education and labor force participation [2,3]. In 2015, the global community reaffirmed commitments to providing universal access to FP services so that women and couples have the opportunity to determine their desired number and spacing of children [4]. This commitment remains central to the attainment of Sustainable Development Goal 3.7 by 2030 [5]. Despite substantial increases in the number of new FP users, in 2022 56% of women in sub-Saharan Africa had their demand for FP satisfied, defined as the number of women of reproductive age who are using modern contraception and do not desire pregnancy in the next two years or who have not had a pregnancy or birth in the past two years that they wished to delay or prevent [4,6]. In Tanzania and many other countries, the lowest satisfied demand for FP is concentrated in rural areas, predominantly among women who lack formal education, and women in the lowest wealth quintile [7–9].

Recognizing the major health impacts of addressing unsatisfied demand for FP, the World Health Organization and the Tanzanian National Family Planning Costed Implementation Plan have highlighted the importance of addressing sociocultural and community barriers in order to decrease unmet need for modern contraception [4,9]. Uncertainty about compatibility of FP with Islamic religious beliefs contributes to poor acceptance and hinders uptake of modern contraceptive methods among some Muslim communities in Tanzania and other parts of East Africa [10–12]. Moreover, a multilevel analysis using data from 29 demographic and health surveys across sub-Saharan Africa reported that Muslim women were 47% less likely to use modern contraception than Christian women, and postulated that this might have been due to religious beliefs [13]. Recent work in northwest Tanzania documented that male and female Muslim leaders widely believed that spacing births is promoted in Islamic scriptures, but were less certain about whether modern contraception was acceptable in their faith tradition [10]. Religious leaders are trusted, influential members of Tanzanian society whose teaching about a variety of health topics can impact health behavior of their communities [14–18]. Building upon prior evidence from Tanzania, Kenya and Nigeria [15–20], we hypothesize that partnerships with Muslim religious leaders to educate Muslim communities about FP could improve FP knowledge in those communities and may ultimately decrease unsatisfied demand.

A recent cluster-randomized trial among Christian religious leaders in northwest Tanzania demonstrated that an educational intervention about FP led to an increased uptake of contraceptives compared to communities whose religious leaders did not receive the intervention [18]. The robustness of these data suggested that implementing an analogous FP educational intervention for Muslim religious leaders may offer an opportunity to bolster knowledge about FP in the context of both faith and health. Using recent qualitative data from the same region [10], this evidence-based FP curriculum for Christian religious leaders was adapted for implementation among Muslim religious leaders. Due to the previous curriculum’s reliance on Biblical texts, as well as the importance of incorporating specific Qur’anic texts and ethico-legal Islamic teaching, it was essential that the educational curriculum be substantially revised to fit the Islamic context. In this study, following adaptation, the educational seminar was pilot-tested among Muslim religious leaders in a rural community in northwestern Tanzania. The aim of this study was to evaluate the acceptability, appropriateness, and feasibility of the pilot seminar among Muslim religious leaders for future implementation in Tanzanian communities. In-depth interviews sought to explore participants’ perspectives on FP and actions taken in the weeks following the seminar, and to seek suggestions for additional improvements.

## Methods

### Study design and setting

This study utilized a mixed-methods approach to quantify participants’ ratings of the pilot educational seminar’s acceptability, appropriateness, and feasibility, and in-depth interviews to explore participants’ views. Quantitative surveys and in-depth interviews were conducted six weeks after the educational seminar. This study was conducted in Nyanguge, a roadside ward located approximately 40km from Mwanza city in northwestern Tanzania. Muslim male religious leaders and female elders were sampled from two mosques in Nyanguge associated with the National Muslim Council of Tanzania (BAKWATA), a well-established faith-based Islamic organization in Tanzania [21], were invited to attend the educational pilot seminar. Although a variety contraceptives are provided free of charge at governmental health facilities in Tanzania, the country’s most recent Demographic and Health Survey data documented that an estimated 24% of married, reproductive-aged women in this region of Tanzania would like to prevent pregnancy but are not using modern contraception [8].

### Pilot-testing of the educational seminar

Before the pilot seminar, study team members visited both mosques in Nyanguge to provide information about the upcoming educational seminar and to invite the leaders of the mosques to share the information with eligible attendees. The mosques represented two Muslim denominations, Sunni and Shia, and together had over 400 weekly attendees. Those who were eligible to attend the seminar included religious leaders, elders, and lay leaders who attended either mosque and were above 18 years of age. Religious leaders from mosques outside of Nyanguge were excluded from participating in the study given that the focus is on a community-based intervention. The one-day pilot-seminar was held in the community’s most spacious mosque. We used purposive sampling to ask leaders in both mosques to invite other leaders who they felt would be interested and who were also eligible to participate.

The pilot seminar was adapted from a previous version of the FP educational curriculum for Christian religious leaders [18] and from prior qualitative studies conducted among Muslim community members and Muslim religious leaders in the region [10,15]. The seminar included a presentation of data from prior focus group discussions that had been conducted among Muslims to understand prevailing views on FP, a discussion of FP and Islamic ethico-legal and theological principles, and straightforward medical teaching about FP and modern contraceptives, including efficacy, risks, and benefits of each method. Feedback from a previous version of the curriculum prompted us to educate men and women together because prior participants had reported that they appreciated knowing that the other gender was receiving the same information about FP. The adapted seminar included both instructive and open discussion sessions. The seminar was co-taught in Kiswahili by two senior Muslim religious leaders from the Mwanza region, and one obstetrician-gynecologist from Bugando Medical Center in Mwanza. Printed books containing the slides that were used during the seminar were provided to all participants.

### Study tools

Six weeks after the educational seminar, all Muslim religious leaders who attended the seminar were invited to complete a one-page quantitative evaluation assessing three implementation outcome measures as well as participate in in-depth interviews.

Evaluation of implementation outcome measures is essential for assessing how an intervention is being implemented and for understanding how participants perceive the intervention. Outcome measures can identify shortcomings of the implementation process and point to important contextual factors that affect community implementation, potentially including community members’ attitudes, end user behaviors, and structural enablers or barriers [22,23]. To assess these measures regarding the educational seminar provided to religious leaders, this study employed the widely-used psychometric assessment proposed by Weiner and colleagues [22] to measure three implementation outcome measures in the quantitative survey: Acceptability of Intervention Measure (AIM), Intervention Appropriateness Measure (IAM), and Feasibility of Intervention Measure (FIM) (see **S1 File**) [22]. Measures were defined as follows: *Acceptability* was the perception that the seminar was agreeable, satisfactory and appealing; *Appropriateness* reflected perceived fit and compatibility of the seminar; and *Feasibility* was the perception that the seminar can be successfully implemented and carried out in the community [22]. The one-page quantitative survey consisted of four questions for each outcome measure and asked participants to rate their strength of agreement with each statement using a Likert scale that ranged from 1–5 (1 = completely disagree, 2 = disagree, 3 = neither agree nor disagree, 4 = agree, 5 = completely agree). The quantitative survey was adapted for relevance to the FP seminar in English, professionally translated into Kiswahili, and back translated into English for verification. All participants who attended the one-day pilot seminar were invited to complete the anonymous survey which lasted approximately 10 minutes. Survey responses were entered into REDCap, a secure database, and mean composite scores for each statement and for each outcome measure were calculated. Means with standard deviations (SD) were computed for the three implementation outcome measures using R software, version 1.2.1335.

### In-depth interviews and qualitative analysis

Open-ended, semi-structured interviews were conducted in June 2022, and were used to explore participants’ knowledge about FP, perceptions about permissibility of using FP in Islam, and views on the pilot-educational seminar on FP. Participants were asked to refer to and elaborate on their responses to the quantitative survey. Participant sociodemographic data including age and education level were collected at the close of the in-depth interview. Identifying information was not collected during interviews. In-depth interviews were conducted in a private setting in Kiswahili by trained social scientists who were of the same gender as participants, were not members of the study teaching team, and did not live in Nyanguge. Interviews were digitally recorded and lasted approximately 45 minutes.

Audio recordings were transcribed verbatim into Kiswahili and professionally translated into English in Mwanza. Transcripts were imported and analyzed using NVivo 12 (QSR International, Australia). Four study team members independently reviewed three transcripts and developed a set of codes, and at least two team members coded all interviews. Codes were discussed and consensus reached by group process to develop a code sheet that would be used for coding additional transcripts. During coding, *in vivo* codes that were identified were discussed by the group for inclusion. Codes were mapped to the three implementation outcome measures of acceptability, appropriateness, and feasibility. Coding continued until data saturation was reached. Illustrative quotations were selected verbatim to communicate participants’ views. Additional themes related to the seminar’s impact on individuals and community members that were discussed and agreed upon by group process. Reflexivity remains an important aspect of qualitative research and rests on critically recognizing how the authors’ predispositions and perspectives may affect study analysis [24]. All authors were cognizant to adopt as objective a stance as possible during qualitative analysis, specifically regarding the data, participants, and context of the study, and authors discussed emerging themes openly during analysis. No study team members had any affiliation with study participants, and interviewers were independently hired without any biases related the study. All authors agreed on the final themes and illustrative quotes.

### Ethics approval and consent to participate

This study received ethical approvals from research ethics committees at the National Institute of Medical Research (Dar es Salaam, Tanzania) and Weill Cornell Medicine (New York, USA). All participants provided written informed consent prior to enrolling in the study. Participant surveys did not include identifying information. Digital recordings of in-depth interviews that had potentially identifying information were removed during transcription. To preserve participant confidentiality, all original audio recordings were destroyed after study completion.

### Inclusivity in global research

Additional information regarding the ethical, cultural, and scientific considerations specific to inclusivity in global health research is included in the Supporting Information (**S2 File**).

## Results

A total of 48 Muslim religious leaders and elders attended the one-day educational seminar on FP. In June 2022, six weeks after the pilot seminar, all 48 Muslim religious leaders who attended the seminar participated in in-depth interviews, including imams and self-identified male and female leaders in the community. Twenty-six women (54%) and 22 men participated. The median age was 41.5 [IQR 30–59.3] years. Over half (62.5%) reported being married and the median number of children was 2 [IQR 1–5.3]. Participants reported attending a median of 2 [IQR 1–15.8] religious services per month (**Table 1**).

**Table 1:** Sociodemographic characteristics of 48 Muslim religious leaders.

Characteristics	Total Leaders (N = 48)	Women Leaders (n = 26)	Men Leaders (n = 22)
**Age (years)**			
**18–30**	12 (25.0%)	7 (26.9%)	5 (22.7%)
**31–45**	15 (31.3%)	7 (26.9%)	8 (36.4%)
**46–60**	9 (18.8%)	4 (15.4%)	5 (22.7%)
**61 and above**	12 (25.0%)	8 (30.8%)	4 (18.2%)
**Median age (IQR)**	41.5 [30–59.3]	42 [30.3–61.3]	40.5 [30.8–54.8]
**Level of education (%)**			
**No education**	12.5	23.1	0
**Primary**	64.6	61.5	68.2
**Secondary**	18.8	11.5	27.3
**Higher**	4.2	3.8	4.5
**% Married**	62.5	50	77.3
**Median number of children (IQR)**	2 [1–5.3]	3 [2–5]	2.5 [1–5.5]
**Median number of religious services attended per month (IQR)**	2 [1–15.8]	1 [0–0]	24.5 [7–35]
**% reporting ever using modern contraception**	54.2	53.8	36.4
**Contraceptives used (%)** ^**a**^			
**Oral Contraceptive Pills**	10.4	28.6	12.5
**Intrauterine Device**	4.2	7.1	12.5
**Injection**	10.4	28.6	12.5
**Implant**	16.7	35.7	37.5
**Condom**	2.1	0	12.5

^a^ Calculated for those who reported having ever used any modern contraceptive method.

Participants rated the seminar on FP as highly acceptable, appropriate, and feasible, assigning mean scores above 4.5 out of 5 for every statement (**Table 2; S3 File**). The seminar was rated as acceptable by 94% and as appropriate by 98% of participants. Fewer felt that the seminar was feasible, with 3 participants (6%) disagreeing with at least one statement. Our qualitative data illustrate several important themes with participants expanding on reasons for their ratings, as described below.

**Table 2:** Mean composite scores of AIM, IAM, and FIM for assessment of the family planning pilot-tested educational seminar.

Indicator	Mean Composite Score (out of 5 possible points) and SD for each statement given by 48 participants
**Acceptability**	
This seminar about family planning meets my approval.	4.75 (0.48)
This seminar about family planning is appealing to me.	4.83 (0.43)
I like this seminar about family planning.	4.77 (0.56)
I welcome this seminar about family planning.	4.79 (0.54)
**Appropriateness**	
This seminar about family planning seems fitting.	4.65 (0.48)
This seminar about family planning seems suitable.	4.73 (0.45)
This seminar about family planning seems applicable.	4.71 (0.46)
This seminar about family planning seems like a good match for my community.	4.69 (0.59)
**Feasibility**	
This seminar about family planning seems implementable.	4.52 (0.74)
This seminar about family planning seems possible to use here.	4.67 (0.56)
This seminar about family planning seems doable.	4.71 (0.46)
This seminar about family planning seems easy to use.	4.71 (0.62)

Most participants expressed appreciation that the seminar increased their knowledge about the benefits of FP while correcting previous incorrect information. Additional themes emerged related to the effect that the seminar had both on the individuals who attended, and subsequently on those around them as they shared the knowledge that they had gained with others.

### Acceptability

There was general agreement among male and female leaders that the FP seminar was acceptable in their communities, leading to a mean acceptability score of 4.65 out of 5. Participants described having a better understanding of FP as a means of birth spacing and helping couples achieve their desired reproductive goals. They appreciated that the seminar provided them an opportunity to learn about the benefits of FP for health, explaining:

“*the seminar was good, because it also trained people who were not aware of how this FP is*” [Woman, 43 years].

“*[I] didn’t know…FP methods like an implant and pills…I benefited with the education…that a mother’s health is affected when she gives birth randomly*. *Sometimes even losing her life…so I have got an education that a woman must get a resting time about two and a half or three years before giving birth to another child*.*”* [Man, 30 years]

Participants further indicated that the seminar had enhanced their confidence about FP within the Islamic religious context. They reflected on how the seminar provided a new understanding of FP to bolster family health in their community. They stated that incorporation of verses from the Qur’an further increased the seminar’s acceptability:

*“we came and our minds were opened…what I can say, the seminar gave us light [enlightened us]”* [Man, 67 years].:

*“They even talked about the Qur’an verse that talks about [FP] so even for the people who didn’t understand and think that FP is just for the researchers and the government*, *but we discovered that it is there even in the religious books…believers who were there saw that this is a good thing*.*”* [Man, 54 years]

Importantly, many women participants noted that the seminar had been acceptable for opening discussions of women’s problems with men in their community. They explained that the seminar helped to shift men’s prior contraceptive beliefs and addressed gaps in knowledge regarding women’s use of FP methods. Even further, women appreciated that the seminar fueled men’s understanding of how FP could preserve women’s health and enable their children to flourish. One woman stated:

“*Men heard the problems women are going through… they are being taught, because when women tell them they do not take it seriously… [but through teaching at the seminar] they can see the importance and see the problems women go through.”* [Woman, 30 years]

Some participants noted the importance of men and women learning together in the same space. They explained the strength of learning together increased men’s awareness and encouraged conversations about FP use:

*“From my thoughts…it was a good one because a lot of people, we did not know about FP. Women and men had to gather together…you see, they did not separate us; we were at the same group. Personally, I saw it as a right thing.”* [Woman, 30 years]

In contrast, others questioned the acceptability of men and women learning in the same space. They explained that both men and women would be more inclined to ask questions and engage in meaningful dialogue about FP if seminar sessions were held separately. One participant stated:

*“You can find that in that seminar maybe there is a father with his in-laws, there are children, there are other questions that are asked, and it is embarrassing. That is why…if another one happens, we should be separate.”* [Woman, 37 years]

A number of participants mentioned feeling validated because educators had prioritized teaching their community about FP. They felt that the seminar’s efforts to visit Nyanguge and incorporate Islamic religious teachings showed respect and value for their community and religious beliefs:

“*…bringing this education here, here in Nyanguge, I really liked it*” [Man, 35 years].

“*What attracted me is that when you came*, *that you are our visitors*, *and you came to educate us and recognize us…we have been remembered*.*”* [Woman, 59 years]

Another participant especially appreciated that the seminar was co-led by Sheikhs and health professionals, resulting in integration of medical information with verses from the Qur’an:

“*There were researchers, there were religious leaders, there were health professionals, and the main topic was FP… another thing that touched me was the religious leaders supporting the use of FP. They also had lines from the Qur’an, it describes the use of FP*.” [Woman, 32 years].

### Appropriateness

Appropriateness of the seminar was assigned a mean score of 4.69 out of 5, with participants expressing that the seminar appropriately addressed needs for improved health and well-being of women and children in their communities. Both men and women emphasized that the seminar appropriately explained the positive effect of FP on the stability of families, including the ability to feed, educate and care for their children, stating:

“*[the seminar] is appropriate with the community and us [religious leaders] too because it helps us to educate people who did not know more. Protecting their health and having developments in their families*.” [Woman, 29 years]

“*I personally saw that it was appropriate*, *because the biggest problem is…you find a parent has a huge load because the children are overwhelming you*. *Now their health…the plan to provide them their school needs*, *to dress*, *eat*… *[FP] is just someone creating an environment to be able to satisfy their needs*.*”* [Man, 48 years]

Participants also reported that the seminar equipped them with knowledge to convey this much-needed FP information to family members and neighbors in their community. One participant stated:

“*This seminar impressed me. I even wished to go around and educate other people…there are my fellow women that I do business with… when we sit while resting, we start talking about it: “My fellow women you know it is a great thing to use FP*!” [Woman, 50 years]

Many participants also viewed the seminar as appropriate because of its potential to reshape men’s prior views about FP. Female participants explained that men are often key decision makers in the household, yet some men’s disapproval of FP fostered misunderstandings in relationships and fueled covert contraceptive use. Participants recounted that the seminar had appropriately addressed this major need:

“*I am very grateful that [the seminar] has brought inspiration to men… you gave a good seminar. There are some men who have changed… there [in the seminar] you set them right.”* [Woman, 35 years]

*“The community can understand*, *including men who were not aware of this compared to women*. *They [men] don’t understand or even know the outcome*. *And that is why you may find that there are some misunderstandings in the house*. *So this seminar has come to educate [us about] a lot of things*.*”* [Man, 35 years]

Participants also shared their improved ability to discuss FP with partners after the seminar. Many described a changed view of FP no longer as only a “woman’s affair” but as a topic that couples could discuss. As one man explained, the seminar enabled discussion of a topic that would have been viewed suspiciously by his wife:

“*It is good because, if I hear about it [from elsewhere] and tell my wife, she will be like, ‘Where have you heard that? You have gone out there and bring those things in the house, do not talk about them.’… you have gathered us all. Everyone is getting out of the seminar telling their partners that, you see? I told you that we should use FP and you refused, you see now?”* [Man, 67 years]

Others spoke of how seminar helped them to build trust, and promoted open communication among their partners:

*“It will strengthen their love, between the men and women… But also to reduce fights and misunderstandings in the family…they will go and speak at home that ‘you were refusing for me to use FP [by saying that if I use it] I will get cervical cancer while it’s not true’… the knowledge is important for them so that those misunderstandings…can be eliminated.”* [Woman, 33 years]

Further, participants reflected on the appropriateness of educators who came from outside of the community (in this case, from Mwanza city) to provide information about FP to men in their community. Several suggested that men had been more open to learning about FP from the seminar than they had been to learning from their female partners:

“*You know a wife can take a word [about FP] to a husband and there will be two things, to be accepted or rejected…and he will not want to hear anything. But when the seminar comes, then [he might stop being hesitant].”* [Man, 50 years]

“*Men had already ignored these FP things*. *Now I see that it is appropriate for them*, *and they have already started getting the education*…*The one thing that attracted me about the seminar [was discussions about] FP so that even men can be careful and know that… [women] go through a lot… You might get that when you tell a man [you need a break] he refuses…so after getting that seminar you find that they are somehow getting educated*.” [Woman, 30 years]

### Feasibility

Regarding feasibility, participants assigned a mean score of 4.65 out of 5. Most expressed views that the seminars would be feasible to expand and implement in other communities:

“[The seminar] *can be implemented… When one gets the knowledge and sees the benefits it can be a catalyst for one to get into FP… or understanding of the matter*.” [Woman, 33 years]

“*These seminars are educating us on this good plan*. *We accept it and received that seminar well*, *and luckily you also gave us books…it should continue and we should work on it within our community and to our grandchildren*, *for example*, *we educate our grandchildren on that*.*”* [Man, 62 years]

Many participants also explained the feasibility of educating and disseminating the FP information they learned within their community, explaining:

“*When experts of FP come and educate people in the society, be it Muslims or Christians, then it will be feasible a hundred percent because you will have given them evidence already. Second you will have shown them that it is not a loss. It has benefits and you explained the benefits already so it will be done very well… you [will] give them knowledge*.” [Man, 22 years]

Others reflected on the importance of being trusted ambassadors in their community to promote FP education, expand awareness, and encourage conversations about FP use. Participants acknowledged that many community members were eager to learn and that they themselves were thrilled about the opportunity to teach their community about FP:

*“Especially after having the seminar for the first time…we realized its importance…we become ambassadors for the education we got, we become ambassadors even to our fellows so they [can] use it.”* [Man, 55 years]

Notably, feasibility was the measure that received lowest mean scores during evaluation. A number of participants were not certain whether community members would be willing to learn about FP, and therefore were not sure that the seminar would be implementable in their community. A woman who said she only partially agreed that the seminar would be feasible stated that:

“*There is someone who will tell you I cannot go [to the seminar]. Because I cannot work on what they are talking about.”* [Woman, 29 years].

Another participant described her perception that some community members could flatly reject FP education as it goes against their Islamic religious beliefs. She explained that the historical absence of such practices within the community would raise questions about its use now, stating that *“when you tell them that you were in a FP seminar*, *they ask if nowadays Muslims use FP*. *They ask why in the past we didn’t use it*.*”* [Woman, 50 years]. She further explained that she nevertheless had endeavored to educate these community members about the benefits of FP:

*“This seminar came to give me like an excitement to know more. That means after getting out here I went to the community that surrounds me. Without caring if it is a Muslim or Christian or who does not have religion at all. So as to give them the understanding on family planning, they should use it to help them and uplift their lives*.” [Woman, 55 years]

These data illustrate the tension between religious leaders’ excitement to share knowledge that could alleviate hardship in their communities, and the challenges that they had experienced as they attempted to share this information with some community members. Community members who rejected teaching about FP cited its incompatibility with religious beliefs, although underlying social or cultural factors, more difficult to explicate, may also negatively influence the acceptance and use of FP.

### Benefits of the FP seminar

Participants expressed that the seminar provided them with a renewed understanding of FP in the context of their faith. Many stated that the medical and theological discussions aided in reframing previous beliefs about FP. One man described a question-and-answer session as impactful:

*“We asked questions about cancer, about negative effects. And we also asked other questions on why the period disappears, where does it go? So, the doctor explained to us. I was satisfied.” [Man, 48 years]*

Another participant noted that they were more receptive and open-minded when hearing about FP because the seminar was co-led by Sheikhs and health professionals. They respected that the seminar sessions incorporated verses and stories from the Qur’an which enabled transformation of previous views:

*“I did not take notes the day of the seminar, we were given those papers, I opened and found out that these things are in the Qur’an, it has been written in the Qur’an. Good thing the Sheikh was also there reading about FP, he read from the Qur’an and translated the stories of the prophet that related to FP.” [Man, 22 years]*

One woman shared that her previous perspective on FP had centered on what she had learned growing up, yet the seminar was able to challenge these prior assumptions, she stated:

“*[I learned] those stories that our prophets only bore these children [and that therefore] there couldn’t be this FP… after reaching on this seminar we learnt a lot of things that were under the carpet, that is why we loved it because we discovered a lot of things*” [Woman, 55 years].

### Challenges encountered and recommendations to improve the FP seminar

Participants provided numerous suggestions for improvement. The most frequent suggestion was that these seminars should return to their community and continue to provide education. They suggested increasing the frequency of seminars to encourage community engagement and promote awareness and understanding of FP:

*“I see these seminars that you bring…they should come twice in a month”* [Woman, 22 years].

*“Once you have regular seminars people will be coming and they will be good ambassadors out there”* [Man 30 years].

Both men and women suggested that future seminars should explain the side effects of modern contraceptive methods in greater detail. They felt that this would be a critical improvement to the seminar, and many referenced a general Islamic teaching that forbids putting anything into the body that can cause harm. For some, the question of whether FP was permissible remained incompletely resolved:

*“they did not explain why it affects other people. The reason why other people can experience those effects. So, I didn’t understand why”* [Woman, 29 years].

*“I wasn’t told the effects of using local methods…we were explained that it [modern contraception] has some few effects…but I wanted them to explain to me so deeply so that I can know these things and their effects*.” [Man, 27 years]

The majority of participants cited the importance of increasing the frequency of the educational seminars in their community. Many suggested that the repeated provision of seminars would allow individuals to gain a better understanding of FP and improve community knowledge, which in turn would encourage individuals to see the value of FP. Others suggested that repeated exposure to FP seminars will allow individuals who have fallen behind to catch up.

*“Coming again after two months will encourage that person, it means she will be having a knowledge about it. She will see the value.”* [Man, 26 years]

*“Leaders should not forget to educate the community because if a seminar comes more often people will understand*. *Even a person who was falling back he will come up again*.*”* [Man, 50 years]

Some participants expressed their discomfort participating in the seminar because men and women were in the same space. Although many found the teaching sessions provided to both genders together to be acceptable, suggested that it would be beneficial if women and men could be separated afterwards in order to freely engage in conversations regarding their experiences and ask questions. Participants shared:

*“Some people were shy, because men, women…were there… people were afraid to ask questions with that environment.” [Woman, 42 years]*

“*It will be great if women will be alone and men alone. There are other women things to talk about. And there are other men things to talk about.” [Woman, 50 years]*

*“Women should stay with their fellow women*, *and men should stay with their fellow men*. *Because in the seminar there are different people… there might be a mother who would want to ask a very personal question… if she is with other women then she will talk about it all*.*” [Man*, *50 years]*

Participants highlighted the importance of strengthening the resource materials provided during the educational seminar. Many suggested that clear visual aids, including accurate anatomical illustrations, would provide individuals with a straightforward understanding of contraceptive mechanisms and potential outcomes of their use. Some reflected on the need for comprehensive educational materials that go beyond stating the benefits and side effects of contraceptives and include additional detail on the reasons for differing contraceptive efficacies and side effects.

*…“you can teach someone by using writings or drawings. That if it is from here, it will cause a certain thing, because after entering a certain place, it will go to cause something somewhere.” [Woman, 29 years]*

*“The reason why people can experience those effects*, *I didn’t understand why*, *I didn’t understand well*. *Why does it cause problems*?… *you said that it is good to use*, *but why does it bring some challenges*?*…I wanted to understand more…I wanted someone to explain to me the reasons why it works for some people and affects others*.*” [Woman*, *29 years]*.

Importantly, older participants recognized the importance of engaging adolescents and young adults in FP educational seminars. They suggested that the youth are often those in greatest need of FP and FP knowledge. They emphasized the importance of reaching out to young adults who are starting families, ensuring that they receive the necessary information and support to make informed decisions about their reproductive health.

*“Male and female youth. Youth are more active in getting FP…. (they are) mostly users.” [Woman, 59 years]*

“*What I think should be added in the next meetings, girls and youths should be included….people with the age of eighteen years old, who are able to start a family. She is supposed to be in the training.” [Woman, 43 years]*

*“Young people are supposed to be the majority because they are the targets*, *we are only elders to motivate*.*” [Man*, *73 years]*

## Discussion

Our data demonstrate that Muslim religious leaders who attended a pilot-test version of an educational seminar about FP reported feeling inspired and equipped to bring messages about FP to their communities. Leaders described subsequently using this education to teach community members, both inside and outside of the mosque. Our quantitative and qualitative data suggest that the FP seminar was viewed as enlightening and compatible with the Islamic faith, with the large majority of participants rating the seminar as highly acceptable, appropriate, and feasible for implementation in their communities. Additional themes that emerged in qualitative data were that religious leaders felt valued, experienced improved communication with partners, and often had reframed their prior views on FP. These data, including our and others’ previous work [10,14–20] illustrate the high potential of these trusted religious leaders as partners who can address unsatisfied demand for FP in their communities.

Religion remains a critical aspect of the social and cultural fabric and is firmly intertwined with day-to-day practices for a majority of Tanzanians. Our work complements several prior studies that have demonstrated a positive influence of faith-based organizations and religious leaders in promoting FP use to meet unsatisfied demand in sub-Saharan Africa [25,26]. In a recent ethnographic study, Rafiq and colleagues examined Muslim religious leaders’ role as FP advocates in southwestern Tanzania and highlighted their positions as influential, respected leaders who were able to guide and reconcile community concerns about the use of FP [26]. Our data furthers findings from work with Muslim communities in Kenya, Ethiopia, and Uganda [27–29], underscoring the importance of providing accessible, accurate medical information to increase community knowledge about the efficacy, benefits and side effects of FP methods. Qualitative data demonstrate religious leaders’ renewed understanding of FP in the context of their faith, and the acceptability of disseminating FP messages that align with Islamic religious beliefs. They expressed that the seminar gave them an informed perspective of the benefits of FP to alleviate poverty, improve the health and well-being of women, and encourage partner communication. Moreover, our results reinforce the concept that FP teaching provided by trusted religious leaders can bolster knowledge about FP in communities, enabling women and couples to plan the spacing of their children and promoting the health of families and communities.

Our results illustrate the potential of this intervention to transform perspectives of FP from a “woman’s issue,” the status quo described by many participants prior to the seminar, to one decided by couples. Several studies in sub-Saharan Africa have posited that internalized gender and religious norms positioning men as the head of the household and as key decision-makers can highly influence women’s non-use of FP [30–32]. Further, a recent systematic review of studies in Nigeria [33] reported that partner objection was the main driver of the low uptake of FP. The authors noted that involving men in discussions about FP could improve spousal communication and foster joint decision making about FP. Our qualitative data similarly indicate the importance of increasing men’s knowledge about FP, with many expressing a renewed understanding of the health and economic benefits of FP. In addition, a majority of participants reported that the seminar had provided an opportunity, often for the first time, to discuss and decide jointly about FP with their partner. This effect was also observed in an FP educational intervention among Christians and was linked with increased self-efficacy to understand and decide about FP [18]. We posit that the example of male religious leaders engaging in this topic during our educational seminar may be instrumental in facilitating men’s engagement among community members.

Qualitative data obtained in this study provide key action points as we strive to increase this pilot curriculum’s acceptability, appropriateness, and feasibility in Muslim communities. While many participants expressed optimism about the feasibility of the educational, others worried that community members might be hesitant to learn about FP due to perceived conflicts with engrained cultural and religious beliefs, particularly the widely held norm that prizes having large families [34]. Recognizing this, it will be critical to discuss the relationships between Islamic religious values, traditional norms, and health outcomes in future FP educational seminars. A prior trial in Tanzania among Christian religious leaders [18] has underscored the potential impact that similar work among trusted Muslim religious leaders could potentially have for educating communities about FP. Obtaining Muslim leaders’ perspectives on a next iteration of this seminar that includes joint teaching sessions for men and women followed by time in which they can ask questions separately will be invaluable. To further strengthen feasibility, we plan to incorporate Muslim leaders’ suggestions to use clear visual representations, explain side effects in greater detail, and provide post-seminar check-in/mentorship meetings to touch base and answer any questions that have arisen. Together, we anticipate that these adaptations to the pilot-tested version of this seminar could be beneficial to clarify points of confusion and strengthen the likelihood of intervention feasibility and sustainability.

Within the Islamic tradition, uptake and practice of interventions such as FP need to be deliberated according to a well-established five-point ethico-legal framework. This involves consideration of whether acts are obligatory, recommended, permissible, reprehensible, or forbidden. The first category refers to religious obligations such as offering of five daily prayers and fasting. The second refers to acts that are good to perform but not required, such as additional prayers for piety. The third category encompasses the majority of activities and decision making, where they are largely neutral or permissible unless there is clear instruction for prohibition [35]. Evidence based interventions like FP fall into the third category of permissibility [36]. Such a rubric can lend further insights into how educational programs and associated interventions can be framed to enable Muslim communities to envisage how FP can be embedded successfully and in coherence within their religious beliefs. Given some participants’ concerns that FP may not be acceptable because of potential side effects, we plan to incorporate this rubric and further discussion of the risks and benefits of both closely spaced births and specific contraceptives into future versions of this curriculum.

## Limitations

This study benefited from a mixed-methods approach that provided both comprehensive analyses of the acceptability, appropriateness and feasibility of the seminar and contextualized insights into participants’ perspectives on the seminar about FP. This study was subject to certain limitations. As this study utilized an exploratory mixed-methods sequential design, it was reliant on participant report, and although interviewers were not people who had provided the educational seminar, it is possible that some participant responses were limited by social desirability bias. Additionally, the small sample size makes it difficult to draw reliable conclusions about the three outcome measures assessed after the dissemination of the pilot seminar and about how generalizable participants’ views of the seminar are. Future evaluations could benefit from seeking participant feedback not only 6 weeks after the seminar, but over a longer time frame, to capture community perspectives on FP and identify community adoption of various FP methods.

## Conclusion

Findings from our pilot-study provide concrete steps to enhance the feasibility of providing an educational seminar about FP for Muslim religious leaders that could have meaningful effects on community knowledge of FP. From the first iteration of this pilot-study, our data indicate that religious leaders’ broadened perspectives about FP in the context of their faith galvanized them to teach their communities, demonstrating strong potential for wide reach and broader implementation. This work may play a pivotal role in reshaping future FP programs to address unsatisfied demand not only among Muslim communities in Tanzania, but more broadly in sub-Saharan Africa.

## Supporting information

S1 File Psychometric assessment implementation outcome measures. (DOCX)

S2 File Inclusivity in global research. (DOCX)

S3 File Results of psychometric assessment implementation outcome measures. (XLSX)

## References

[1] United Nations. 2020. World Family Planning. https://www.un.org/en/development/desa/population/publications/pdf/family/WFP2017_Highlights.pdf. Accessed 15 Jan 2023.

[2] Kraft JM, Serbanescu F, Schmitz MM, Mwanshemele Y, Ruiz C. AG, Maro G, et al. Factors Associated with Contraceptive Use in Sub-Saharan Africa. *J Women’s Heal*. 2022; 31(3): 447–57. doi: 10.1089/jwh.2020.8984 34129385 PMC8972023

[3] Haakenstad A, Angelino O, Irvine CMS, Bhutta ZA, Bienhoff K, Bintz C, et al. Measuring contraceptive method mix, prevalence, and demand satisfied by age and marital status in 204 countries and territories, 1970–2019: a systematic analysis for the Global Burden of Disease Study 2019. *Lancet*. 2022; 400(10348): 295–327. doi: 10.1016/S0140-6736(22)00936-9 35871816 PMC9304984

[4] United Nations. World Family Planning 2022: Meeting the changing needs for family planning: Contraceptive use by age and method. United Nations. 2022. https://www.un.org/en/development/desa/population/publications/pdf/family/WFP2017_Highlights.pdf. Accessed 4 Mar 2024.

[5] United Nations publication: Department of Economic and Social Affairs. 2022. The sustainable development goals report 2019. https://unstats.un.org/sdgs/report/2019/. Accessed 15 Jan 2023.

[6] Landry M. Unmet need for family planning. World Health Organization. https://www.who.int/data/gho/indicator-metadata-registry/imr-details/3414#:~:text=Women with unmet need are,intentions and their contraceptive behaviour. Accessed 23 Jan 2023.

[7] Kassim M, Ndumbaro F. Factors affecting family planning literacy among women of childbearing age in the rural Lake zone, Tanzania. BMC Public Health [Internet]. 2022;22(1):1–11. Available from: 10.1186/s12889-022-13103-1. 35379226 PMC8981830

[8] Ministry of Health. Tanzania Demorgraphic and Health Survey Indicator Survey (TDHS-MIS) 2015–2016. Vol. 1, Dar es Salaam, Tanzania, and Rockville, Maryland, USA: https://www.dhsprogram.com/pubs/pdf/FR382/FR382.pdf. Accessed Mar 4 2024.

[9] The United Republic of Tanzania: National Family Planning Costed Implementation Plan 2019–2023 (NFPCIP 2019–2023). The United Republic of Tanzania Ministry of Health, Community Development, Gender, Elderly and Children. 2019. https://efaidnbmnnnibpcajpcglclefindmkaj/https://tciurbanhealth.org/wp-content/uploads/2019/07/National-Family-Planning-Costed-Implementation-Plan-2019-2023-INSIDE-%E2%80%A2-F….pdf. Accessed 09 Dec 2022.

[10] Chalem A, Nzali A, Cordeiro AA, Yussuph A, Laizer E, Lupilya G, et al. Perspectives of Muslim Religious Leaders to Shape an Educational Intervention About Family Planning in Rural Tanzania: A Qualitative Study. *Glob Heal Sci Pract*. 2023; 11(1). doi: 10.9745/GHSP-D-22-00204 36853642 PMC9972385

[11] Kenny L, Lokot M, Bhatia A, Hassan R, Pyror S, Dagadu NA, et al. Gender norms and family planning amongst pastoralists in Kenya: a qualitative study in Wajir and Mandera. *Sex Reprod Heal Matters*. 2022; 30(1). 10.1080/26410397.2022.2135736. PMC9704065 36416930

[12] Massenga J, Noronha R, Awadhi B, Bishanga D, Safari O, Njonge L, et al. Family planning uptake in Kagera and Mara regions in Tanzania: A cross-sectional community survey. *Int J Environ Res Public Health*. 2021; 18(4). doi: 10.3390/ijerph18041651 33572305 PMC7916100

[13] Ahinkorah BO, Budu E, Aboagye RG, Agbaglo E, Arthur-Holmes F, Adu C, et al. Factors associated with modern contraceptive use among women with no fertility intention in sub-Saharan Africa: evidence from cross-sectional surveys of 29 countries. *Contracept Reprod Med*. 2021; 6(1). doi: 10.1186/s40834-021-00165-6 34332644 PMC8325798

[14] Downs JA, Mwakisole AH, Chandika AB, Lugoba S, Kassim R, Laizer E, et al. Educating religious leaders to promote uptake of male circumcision in Tanzania: a cluster randomised trial. Lancet. 2017;389(10074):1124–32. Available from: doi: 10.1016/S0140-6736(16)32055-4 28214093 PMC5364327

[15] Sundararajan R, Yoder LM, Kihunrwa A, Aristide C, Kalluvya SE, Downs DJ, et al. How gender and religion impact uptake of family planning: Results from a qualitative study in Northwestern Tanzania. BMC Womens Health. 2019;19(1):1–10. 31331306 10.1186/s12905-019-0802-6 PMC6647140

[16] Lambert VJ, Kisigo GA, Nzali A, Laizer E, Paul N, Walshe L, et al. Religious Leaders as Trusted Messengers in Combatting Hypertension in Rural Tanzanian Communities. Am J Hypertens. 2021;34(10):1042–8. doi: 10.1093/ajh/hpab080 34022044 PMC9214778

[17] Aristide C, Mwakisole A, Mwakisole N, Emmanuel M, Laizer E, Kihunrwa A, et al. Design and pilot testing of a church-based intervention to address interpersonal and intrapersonal barriers to uptake of family planning in rural Tanzania: A qualitative implementation study. BMJ Sex Reprod Heal. 2020;46(3):226–33. doi: 10.1136/bmjsrh-2019-200505 31937520 PMC7392489

[18] Mwakisole AH, Lambert VJ, Nzali A, Aristide C, Laizer E, Cordeiro AA, et al. Articles Partnerships with religious leaders to promote family planning in rural Tanzania: an open-label, cluster randomised trial. Lancet Glob Heal. 2023;11(12):e1943–54. 10.1016/S2214-109X(23)00453-9. 37973342

[19] Bormet M, Kishoyian J, Siame Y, Ngalande N, Erb K, Parker K, et al. Faith Based Advocacy for Family Planning Works: Evidence from Kenya and Zambia. Glob Heal Sci Pract. 2021; 9(2): 254–63. doi: 10.9745/GHSP-D-20-00641 34111021 PMC8324188

[20] Adedini SA, Babalola S, Ibeawuchi C, Omotoso O, Akiode A, Odeku M. Role of religious leaders in promoting contraceptive use in Nigeria: Evidence from the Nigerian Urban reproductive health initiative. *Glob Heal Sci Pract*. 2018; 6(3): 500–514. doi: 10.9745/GHSP-D-18-00135 30287529 PMC6172128

[21] The National Muslim Council of Tanzania. BAKWATA. 2022. https://bakwata.or.tz/. Accessed 15 Dec 2022.

[22] Weiner BJ, Mettert KD, Dorsey CN, Nolen EA, Stanick C, Powell BJ, et al. Measuring readiness for implementation: A systematic review of measures ‘ psychometric and pragmatic properties. 2020. doi: 10.1177/2633489520933896 37089124 PMC9924280

[23] Weiner BJ, Lewis CC, Stanick C, Powell BJ, Dorsey CN, Clary AS, et al. Psychometric assessment of three newly developed implementation outcome measures. Implement Sci. 2017;12(1):1–12. 28851459 10.1186/s13012-017-0635-3 PMC5576104

[24] Olmos-vega FM, Stalmeijer RE, Varpio L, Kahlke R, Stalmeijer RE, Varpio L, et al. A practical guide to reflexivity in qualitative research: AMEE Guide No. 149. Med Teach [Internet]. 2023;45(3):241–51. Available from: doi: 10.1080/0142159X.2022.2057287 35389310

[25] Ruark A, Kishoyian J, Bormet M, Huber D. Increasing family planning access in Kenya through engagement of faith-based health facilities, religious leaders, and community health volunteers. *Glob Heal Sci Pract*. 2019;7(3):478–90. doi: 10.9745/GHSP-D-19-00107 31558602 PMC6816806

[26] Rafiq MY, Wheatley H, Salti R, Shemdoe A, Baraka J, Mushi H. “I let others speak about condoms:” Muslim religious leaders’ selective engagement with an NGO-Led family planning project in rural Tanzania. *Soc Sci Med*. 2022; 293:114650. https://linkinghub.elsevier.com/retrieve/pii/S0277953621009825. doi: 10.1016/j.socscimed.2021.114650 34915242

[27] Kenny L, Bhatia A, Lokot M, Hassan R, Hussein Aden A, Muriuki A, et al. Improving provision of family planning among pastoralists in Kenya: Perspectives from health care providers, community and religious leaders. *Glob Public Health*. 2022;17(8):1594–610. doi: 10.1080/17441692.2021.1944263 34182886

[28] Tigabu S, Demelew T, Seid A, Sime B, Manyazewal T. Socioeconomic and religious differentials in contraceptive uptake in western Ethiopia: A mixed-methods phenomenological study. BMC Womens Health. 2018;18(1):1–10. 29871621 10.1186/s12905-018-0580-6 PMC5989360

[29] Nanvubya A, Wanyenze RK, Abaasa A, Nakaweesa T, Mpendo J, Kawoozo B, et al. Evaluating the effectiveness of enhanced family planning education on knowledge and use of family planning in fishing communities of Lake Victoria in Uganda: a randomized controlled trial. *BMC Health Serv Res*. 2022;22(1):1–12. 10.1186/s12913-022-07898-3. 35421987 PMC9012015

[30] Hoyt J, Krishnaratne S, Hamon JK, Boudarene L, Chantler T, Demissie SD, et al. “As a woman who watches how my family is… I take the difficult decisions”: a qualitative study on integrated family planning and childhood immunisation services in five African countries. *Reprod Health*. 2021;18(1):1–13. 10.1186/s12978-021-01091-1. 33588879 PMC7885443

[31] Koffi TB, Weidert K, Bitasse EO, Mensah MAE, Emina J, Mensah S, et al. Engaging men in family planning: Perspectives from married men in Lomé, Togo. *Glob Heal Sci Pract*. 2018;6(2):316–27. 10.9745/GHSP-D-17-00471. PMC6024630 29743188

[32] Kriel Y, Milford C, Cordero J, Suleman F, Beksinska M, Steyn P, et al. Male partner influence on family planning and contraceptive use: Perspectives from community members and healthcare providers in KwaZulu-Natal, South Africa. *Reprod Health*. 2019;16(1):1–15. 10.1186/s12978-019-0749-y 31238960 PMC6593556

[33] Akamike IC, Okedo-alex IN, Eze II, Ezeanosike OB, Uneke CJ. Why does uptake of family planning services remain sub-optimal among Nigerian women? *Contracept Reprod Med*. 2020;5(30):1–11. 10.1186/s40834-020-00133-6. 33292842 PMC7603738

[34] Alomair N, Alageel S, Davies N, Bailey J V. Factors influencing sexual and reproductive health of Muslim women: A systematic review. *Reprod Health*. 2020;17(1):1–15. 10.1186/s12978-020-0888-1. 32138744 PMC7059374

[35] Al-Bar MA, & Chamsi-Pasha H. Contemporary bioethics: Islamic perspective. *Springer Nature*. 2015. (p.267). 10.1007/978-3-319-18428-9. 29809384

[36] Omran AR. Family planning in the legacy of Islam. 1992. 10.4324/9780203167977.

