# Supplementary material for: Evaluation of a pilot family planning educational seminar and subsequent attitudes towards family planning among Muslim communities in Tanzania
Source: PLoS One. 2025 Feb 7;20(2):e0315410. doi: 10.1371/journal.pone.0315410 (PMC11805577; doi:10.1371/journal.pone.0315410)
Supplement: S1 File — (DOCX) [file pone.0315410.s001.docx]

**Supplemental File 1**

**S1 File:**

**Acceptability Intervention Measure (AIM), Intervention Appropriateness Measure (IAM), and Feasibility of Intervention Measure (FIM) for Assessment of Family Planning Seminar.**

GENERAL INSTRUCTION: Place a mark in one box for each category to show how you feel about each statement.

Acceptability

|  | **Completely disagree** | **Disagree** | **Neither agree nor disagree** | **Agree** | **Completely agree** |
| --- | --- | --- | --- | --- | --- |
| This seminar about family planning meets my approval. |  |  |  |  |  |
| This seminar about family planning is appealing to me. |  |  |  |  |  |
| I like this seminar about family planning. |  |  |  |  |  |
| I welcome this seminar about family planning. |  |  |  |  |  |

Appropriateness

|  | **Completely disagree** | **Disagree** | **Neither agree nor disagree** | **Agree** | **Completely agree** |
| --- | --- | --- | --- | --- | --- |
| This seminar about family planning seems fitting. |  |  |  |  |  |
| This seminar about family planning seems suitable. |  |  |  |  |  |
| This seminar about family planning seems applicable. |  |  |  |  |  |
| This seminar about family planning seems like a good match for my community. |  |  |  |  |  |

Feasibility

|  | **Completely disagree** | **Disagree** | **Neither agree nor disagree** | **Agree** | **Completely agree** |
| --- | --- | --- | --- | --- | --- |
| This seminar about family planning seems implementable. |  |  |  |  |  |
| This seminar about family planning seems possible to use here. |  |  |  |  |  |
| This seminar about family planning seems doable. |  |  |  |  |  |
| This seminar about family planning seems easy to use. |  |  |  |  |  |

Date of Observation (DD/MM/YYYY): |__|__| / |__|__| / 20 |__|__|

*Adapted from Weiner B et al, Psychometric assessment of three newly developed implementation outcome measures. Implement Sci 2017; 12(1): 108.*
